# Warm-needling acupuncture and medicinal cake-separated moxibustion for hyperlipidemia: study protocol for a randomized controlled trial

**DOI:** 10.1186/s13063-017-2029-x

**Published:** 2017-07-10

**Authors:** Mailan Liu, Qian Zhang, Shan Jiang, Mi Liu, Guoshan Zhang, Zenghui Yue, Qin Chen, Jie Zhou, Yifan Zou, Dan Li, Mingzhu Ma, Guobin Dai, Huan Zhong, Zhihong Wang, Xiaorong Chang

**Affiliations:** 1 Acupuncture and Tuina School, Hunan University of Chinese Medicine, 300 Xueshi Rd., Yuelu, 410208 Changsha, Hunan China; 2 Rural Coordination Center of BC, Vancouver, BC Canada; 3 University of British Columbia, Vancouver, BC Canada; 4 The Third Clinical College of Zhejiang Chinese Medical University, Hangzhou, China; 5 Changchun University of Chinese Medicine, 1035 Boshuo Rd., Jingyue Economic Development District, Changchun, 130117 Jilin Province China

**Keywords:** Hyperlipidemia, Acupuncture, Moxibustion, Warm-needling acupuncture, Medicinal cake-separated moxibustion, Traditional Chinese medicine, Randomized controlled trial

## Abstract

**Background:**

Acupuncture and moxibustion has been widely applied to hyperlipidemia treatment in clinical practice in China, serving as an alternative treatment to statins. Warm-needling acupuncture and medicinal cake-separated moxibustion have been separately reported with potential therapeutic effects on hyperlipidemia treatment in several studies but with limitations in study methodology. Combining these two modalities may provide a more advantageous strategy in treating hyperlipidemia. Therefore, a strict evaluation through well-designed randomized controlled trials (RCT) is necessary to determine their efficacy and safety on hyperlipidemia.

**Methods:**

The study a multicenter, open-label, randomized, stratified, active-controlled, noninferiority trial with two parallel groups. Subjects with hyperlipidemia will be stratified into different groups by risk levels of heart diseases. They then will be instructed to the Therapeutic Lifestyle Change (TLC) diet. Those who have not reached the target lipid level will be randomly assigned to the treatments of either acupuncture and moxibustion or simvastatin with a 1:1 allocation. One hundred and thirty subjects are aimed to be recruited. The duration of intervention for this study will be 12 weeks, followed by another 4 weeks for post-treatment assessment. The primary outcome is percentage change from baseline to the end of the study in low-density lipoprotein cholesterol (LDL-C). Other indicators in lipid change, safety and adherence will also be assessed secondarily. The repeated measures, linear mixed-effects model will be applied to the analysis.

**Discussion:**

Acupuncture and moxibustion could be a potentially effective treatment alternative for hyperlipidemia. A study with careful design is developed to evaluate the efficacy and safety of combined acupuncture and moxibustion, by integrating the traditional Chinese Medicine (TCM) regimens with the standardized Western medicine appraisal approach.

**Trial registration:**

ClinicalTrials.gov, NCT02269046. Registered on 26 September 2014.

**Electronic supplementary material:**

The online version of this article (doi:10.1186/s13063-017-2029-x) contains supplementary material, which is available to authorized users.

## Background

### Background and rationale

Hyperlipidemia refers to a condition caused by abnormal metabolism where low-density lipoprotein-cholesterol (LDL-C), serum total cholesterol (TC), triglycerides (TG), and/or high-density lipoprotein (HDL-C) are above recommended levels. This condition is associated with the deterioration in lifestyle and dietary habits and represents an important risk factor for cardiovascular morbidity and mortality. Hyperlipidemia is a risk factor for atherosclerosis, cardiovascular disease, stroke, and other diseases. Large randomized controlled trials in hyperlipidemia treatment have provided evidence that reducing LDL cholesterol concentration with statins is efficient in both secondary and primary cardiovascular disease (CVD) prevention [1, 2]. As a result, individuals at moderate or high CVD risk are often considered as candidates for lipid-lowering therapy with statins. However, statin therapy can incur significant cost to the society. In addition, statin therapy is often associated with low treatment compliance and high rates of side effects [3, 4].

Acupuncture and moxibustion has been widely applied to hyperlipidemia treatment in clinical practice of China. Thus, an increasing number of studies have explored whether acupuncture and moxibustion could serve as an alternative treatment for subjects with hyperlipidemia. As shown in a meta-analysis, acupuncture solely, compared to statins, has demonstrated a more significant effect on decreasing TG and increasing HDL-C, but no superiority in lowering the LDL-C and TC [5]. Meanwhile, moxibustion, which is often administered with acupuncture in Traditional Chinese Medicine (TCM) practice, also plays an essential role in lipid-lowering by warming meridians and facilitating lipid conversion in the TCM theory. The recent studies have further revealed the biological pathway in lipid-lowering of moxibustion [6–8]. This modality, especially the warm-needling acupuncture (acupuncture with a moxa stick), can enhance microcirculation, adjust the lipid metabolism, and thus lower the blood viscosity [6–8].

Medicinal cake-separated moxibustion, an important kind of moxibustion, applies the acupoints, moxibustion, and traditional Chinese herb in an integrative way. It has gained increasing popularity in the practice of hyperlipidemia treatment and thus been further assessed on its potential impact. Some findings have shown, from a perspective of gene transcription and protein expression, that the medicinal cake-separated moxibustion could prevent the formation of atheromatous plaque by adjusting Toll-Like Receptor (TLR) signaling pathways as well as peroxisome proliferator-activated receptors (PPARs), in order to delay atherosclerosis (AS) formation and stabilize atheromatous plaque [9, 10]. Chang et al. indicated that both the medicinal cake-separated moxibustion and direct moxibustion have a certain protective action on endothelial cells of the aorta in the rabbit of hyperlipidemia [9]. Yue et al. found that herb-partition moxibustion delays the formation of atherosclerosis through the inhibition of TLR4 expression [10]. This has provided a new strategy for the research on AS pathogenesis and prevention. In addition, based on clinical observation and systematic review of TCM literature, the selection of meridians and acupoints potentially having effect on hyperlipidemia treatment, has been studied and identified, which includes ten meridians (five *Yin* and five *Yang* meridians), and five acupoints (Stomach (ST), Spleen (SP), Ren (RN), Bladder (BL) and Pericardian (PC)) [11]. Based on these findings, several clinical studies on assessing medicinal cake-separated moxibustion have been undertaken, testing different acupoint prescriptions, medicinal-cake ingredients, treatment duration, etc., in attempt to identify an effective and standardized regimen [6, 12–16]. Most of these studies have shown possible therapeutic effects of the medicinal cake-separated moxibustion on hyperlipidemia, superior to the placebo or noninferior to statins.

In general, acupuncture and moxibustion is shown to be possibly effective in treating hyperlipidemia, with lower cost and fewer serious adverse events [17, 18]. However, due to the lack of robust study design and assessment methodology in existing clinical studies, the findings should be interpreted with caution. According to previous attempts on identifying an optimal regimen of acupuncture and moxibustion for treating hyperlipidemia, the warm needling acupuncture along with medicinal cake-separated moxibustion seems to be a modality that successfully combines the advantages of both acupuncture and moxibustion. This is worth further exploring to warrant its therapeutic effects [12, 15, 16]. So far, very few studies on this combined intervention are available. Hence, there is a need for a well-designed randomized control trial to validate the efficacy and safety of warm-needling acupuncture along with medicinal cake-separated moxibustion, by comparing it with statins.

### Hypotheses and objectives

#### Hypotheses

- Warm needling acupuncture and medicinal cake-separated moxibustion (short as “acupuncture and moxibustion” in the following text) is noninferior to active control, in subjects with hypercholesterolemia, on
  - ° percent change of LDL-C
  - ° absolute change of LDL-C
  - ° percent change of HDL-C
  - ° percent change of TC
  - ° percent change of TG
  - ° rate of subject achieving LDL-C goal
- Acupuncture and moxibustion is superior to active control, in subjects with hypercholesterolemia, on safety and tolerability
  - ° Adherence

#### Primary objectives

To evaluate the effect of 12 weeks of acupuncture and moxibustion compared with active control, on percentage change from baseline in low-density lipoprotein cholesterol (LDL-C) among those with hyperlipidemia.

#### Secondary objectives

- To assess the effects of 12 weeks of acupuncture and moxibustion, compared to active control, in subjects with hypercholesterolemia, on
  - ° the absolute change in LDL-C,
  - ° the percent change in high-density lipoprotein cholesterol (HDL-C),
  - ° the percent change in total cholesterol (TC)
  - ° the percent change in triglyceride(TG)
  - ° the rate of subjects achieving LDL-C goal.
- To evaluate the safety and tolerability of acupuncture and moxibustion, given for 12 weeks, in subjects with hypercholesterolemia
- To evaluate the adherence of acupuncture and moxibustion

### Trial design

This is a multicenter, open-label, randomized, stratified, active-controlled, noninferiority trial with two parallel groups. Randomization will be performed with a 1:1 allocation. Subjects with LDL-C values above the recommended level who meet the inclusion/exclusion criteria will be stratified based on their risk levels of heart disease [19, 20]. Then, they were instructed to follow the NCEP Adult Treatment Panel (ATP) [20] Therapeutic Lifestyle Change (TLC) diet first. After TLC is completed, subjects who have not reached the target lipid level will be randomly assigned to the treatments of either acupuncture and moxibustion or simvastatin. The duration of treatment for this study will be 12 weeks, followed by another 4 weeks for post-treatment assessment.

## Methods

**Fig. 1:**
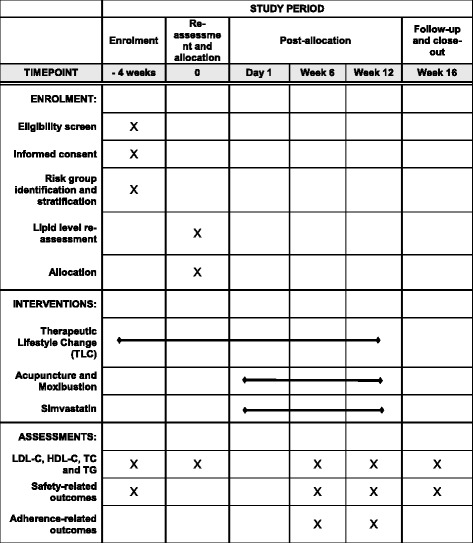
SPIRIT Figure for schedule of enrolment, interventions, and assessments of the AMHRCTstudy

The SPIRIT figure for schedule of enrolment (Fig. 1), interventions, and assessments of this study, and the SPIRIT Checklist are included as Additional file 1 of this protocol.

### Study setting

The study will include four sites in Hunan, China. They are First Affiliated Hospital of Hunan University of Chinese Medicine, Changsha Chinese Medicine Hospital, Yueyang Chinese Medicine Hospital, and Chenzhou Chinese Medicine Hospital. Other sites may be added for participation in the study. Sites that do not enroll any subjects within 3 months of site initiation will be closed. Each site must follow this study protocol; otherwise, it will be closed. The Steering Committee will audit and review the intervention performed in each site, to ensure that they are following this protocol.

### Inclusion criteria

To be included to the study, patients must:

1. Provide informed consent
2. Be male or female ≥18 to ≤75 years of age
3. Have a fasting triglyceride level ≤400 mg/dL (4.5 mmol/L) by central laboratory at screening
4. Have a fasting LDL-C as determined by central laboratory on admission and meeting the following LDL-C values based on risk factor (see Table 2 in Appendix 1) status [19, 20]:
  0–1 Risk Factor Group: LDL-C ≥160 mg/dl
  2+ Risk Factor Group: LDL-C ≥130 mg/dl
  CHD or CHD risk equivalents (see Table 3 in Appendix 1): LDL-C ≥100 mg/dl

### Exclusion criteria

Subjects are excluded from the study if they are diagnosed with one or more of the following conditions:

- Coronary heart disease (CHD) or CHD risk equivalent who are not receiving statin therapy with LDL-C at screening of ≤99 mg/dL
- Heart failure of New York Heart Failure Association (NYHA) class II, III or IV or last known left ventricular ejection fraction <30%
- Cardiac arrhythmia within 3 months prior to randomization that is not controlled by medication
- Myocardial infarction, unstable angina, percutaneous coronary intervention (PCI), coronary artery bypass graft (CABG) or stroke
- Planned cardiac surgery or revascularization
- Type 1 diabetes;
- Newly diagnosed type 2 diabetes (within 6 months of randomization or new screening fasting plasma glucose ≥126 mg/dL (7.0 mmol/L) or HbA1c ≥6.5%), or poorly controlled type 2 diabetes (HbA1c >8.5%)
- Persistent systolic blood pressure (SBP) >160 mm Hg or diastolic BP (DBP) >100 mmHg
- Thyroid stimulating hormone (TSH) < lower limit of normal (LLN) or TSH >1.5 × upper limit of normal (ULN), estimated glomerular filtration rate (eGFR) <30 ml/min/1.73 m^2^, aspartate aminotransferase (AST) or alanine aminotransferase (ALT) >2 × ULN, creatine kinase (CK) >3 × ULN (all at initial screening or at the end of lipid stabilization period(s) by the central laboratory)
- Known major active infection, or major hematologic, renal, metabolic, gastrointestinal or endocrine dysfunction
- Deep vein thrombosis or pulmonary embolism within 3 months prior to randomization

Subjects are also excluded if they have taken any of the following medications for more than 2 weeks in the last 3 months prior to LDL-C screening: systemic cyclosporine, systemic steroids, isotretinoin (e.g., Accutane). Some anticoagulation treatments are also excluded (antiplatelet agents are permitted). Female subjects cannot be pregnant or be breastfeeding and premenopausal women must have to be willing to use at least one highly effective method of birth control during treatment and for an additional 15 weeks after the end of treatment.

### Recruitment

Each investigating site was chosen based on documentation for patient availability. Sites will utilize two main sources for identifying and recruiting potential subjects as described below:

- Incoming hyperlipidemia patients: the four sites in this study are famous for hyperlipidemia treatment in Hunan, China, thus the incoming patients aiming for hyperlipidemia treatments are the primary source for recruitment
- Advertisements: the participation opportunity to this study will be advertised widely in local newspapers and other publications that target hyperlipidemia treatments, in order to recruit enough participants in the recruiting period

### Interventions

All eligible subjects will be instructed to follow the NCEP Adult Treatment Panel (ATP) lifestyle-modification diet first [20]. This approach is designated therapeutic lifestyle changes (TLC). After TLC is completed, subjects who have not reached the target lipid level will be randomly assigned to the treatments of either acupuncture and moxibustion or simvastatin.

#### Therapeutic Lifestyle Change (TLC)

Subjects who meet the inclusion/exclusion criteria will initiate TLC. ATP III recommends a multifaceted lifestyle approach to reduce risk for CHD. Its essential features are:

- Reduced intakes of saturated fats (<7% of total calories) and cholesterol (<200 mg per day) (see Table 4 in Appendix 1 for overall composition of the TLC Diet)
- Therapeutic options for enhancing LDL lowering such as plant stanols/sterols (2 g/day) and increased viscous (soluble) fiber (10–25 g/day)
- Weight reduction
- Increased physical activity

TLC adherence report form is made and sent to subjects to monitor their lifestyle change process. Re-assessment will be conducted after the TLC period.

#### Acupuncture and moxibustion versus simvastatin

According to the re-assessment of fasting lipid after TLC, a decision will be made on whether the lipid level has arrived at the target level (see Table 5 in Appendix 1) and whether subjects should continue with the intervention. For those who have not reached the target lipid level, they will be randomized in equal proportions between “acupuncture” and “simvastatin.”

**Fig. 2:**
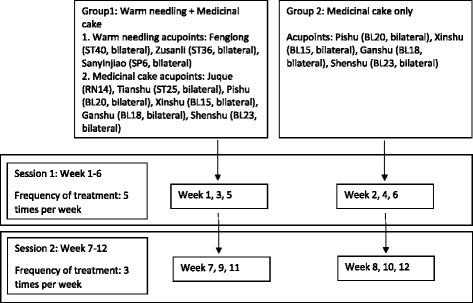
Treatment regimen flowchart. This figure demonstrates the treatment regimen and flowchart for intervention of warm needling and medicinal cake-separated moxibustion
- Simvastatin
  - Dosage: simvastatin (10 mg/day)
  - Administration
    All simvastatin tablets will be stored in the central pharmacy and assigned to each investigator site by a qualified staff. The drug will be administered orally to subjects at the investigator site by a physician or a nurse in accordance with instructions in the medication guides
  - Schedule: 7 days per week for 12 weeks
  - Dosage adjustment:
    there will be no dose adjustments in this study. If, in the opinion of the investigator, a subject is unable to tolerate a specific dose of simvastatin and requires dosage adjustment, that subject will discontinue simvastatin but will continue to return for all other study procedures and measurements until the end of the study

- Acupuncture and moxibustion
  - Rationale for treatment:
    Warm-needling acupuncture and medicinal cake-separated moxibustion will be applied in the study which is guided by the approach of Traditional Chinese Medicine (TCM). The cake-separated moxibustion treatment belongs to the category of indirect moxibustion which applies moxibustion, herb and acupoints together. In this treatment, a drug-cake mixture is allocated upon selected acupoints, and then the lit moxa cone is placed onto the cake. In this case, the moxa-effect enhanced by the active components of the drug cake is allowed to penetrate through the skin and the acupoints.
    Based on the TCM theory that the heart is in charge of the blood circulation and blood vessels, some specific acupoints related to heart meridian and heart are selected, such as the *Shu* acupoint (RN14) and the *Mu* acupoint (BL15) of the heart meridian. BL18, BL20 and BL23 are used to tonify the spleen *Qi*, strengthen the liver *Qi* and also maintain the kidney *Qi*, as well as facilitate the conversion of lipids in the liver. ST40 and ST25, which belong to the stomach meridian and help the transformation function of spleen, can protect and work against hyperlipidemia by assisting the removal of phlegm from the body.
    Additionally, five Chinese herbal medicines are selected, including *Salviae Miltiorrhizae Radix* (*Dan shen*), *Crataegi Fructus* (*Shan zha*), *Curcumae Radix* (*Yu jin*), *Rhei Radix et Rhizoma* (*Da huang*), and *Alismatis Rhizoma* (*Ze xie*). They are able to activate the blood circulation, remove blood stasis and pain, clear away heart fire, remove irritability, nourish the blood and calm down the mind.
    In clinical practice, several studies with small sample sizes have assessed a variety of acupoint prescriptions, medicinal cake ingredients, treatment duration, etc. [12, 16]. Among these studies, Li et al. applied warm needling acupuncture on acupoints such as *Fenglong* (ST40, bilateral), *Zusanli* (ST36, bilateral), etc., once a day for 35 days, over a period of 12 weeks, showing a significant decrease in LDL-C, noninferior to statins [12]. Li et al. applied medicinal cake-separated moxibustion on two groups of acupoints, alternating over a period of 40 days with each group receiving 20 times of intervention [16]. This study demonstrated a remarkable decrease in LDL-C and TC, superior to the effect of a placebo. Meanwhile, Chang et al. have also examined effects of medicinal cake-separated moxibustion with different dosage upon blood fat and hemorheology in patients with hyperlipemia, showing that three moxa cones and five moxa cones both have therapeutic effect [17]. With these studies, so far we know, warm-needling acupuncture and cake-separated moxibustion are both effective for hyperlipidemia, respectively. Together with our previous clinical experiences on treating hyperlipidemia, we selected the studies mentioned above as pilot studies of this trial in determining the procedure course of the intervention.
  - Needling/moxibustion details (demonstrated in Table 1)
  - Treatment regimen:
    Interventions of warm-needling acupuncture and medicinal cake-separated moxibustion will be split into two groups. Group 1 (warm-needling acupuncture and medicinal cake-separated moxibustion) and group 2 (medicinal cake-separated moxibustion only) will alternate by week over the administration period (shown in Fig. 2), in order to avoid fatigue and nonresponse of acupoints due to constant stimulation
  - Practitioners: registered acupuncturists who have completed post-secondary education on acupuncture, practiced acupuncture for more than 3 years, and passed the national qualification exam for Chinese medicine doctors

**Table 1:** Needling and acupuncture details

Warm needling acupuncture (needle + moxa stick)	
Acupoints	*Fenglong* (ST40, bilateral), *Zusanli* (ST36, bilateral), *Sanyinjiao* (SP6, bilateral)
Depths of insertion	*Fenglong* (ST40) for 1.0 to 2.0 cun, *Zusanli* (ST36) and *Sanyinjiao* (SP6) for 1.0 to 1.5 cun (“cun” is a traditional Chinese measure using the width of a person’s thumb at the knuckle, whereas the width of the 2 forefingers denotes 1.5 cun and the width of 4 fingers (except the thumb) side-by-side is 3 cuns. Therefore, 1 cun may vary from person to person)
Needle stimulation	Manual manipulation
Responses elicited	*De qi* sensation
Needle retention time	30 min
Needle specifications	Sterile single-use acupuncture needles of 25–40 mm in length and 0.30 mm in diameter; manufactured by Suzhou Medical Supplies Co., Ltd., Suzhou, China
Moxibustion specifications	Small moxa stick, made of mugwort, with 1.5 cm length; manufactured by Suzhou Medical Supplies Co., Ltd., Suzhou, China
Manipulation	(1) locate and sterilize the acupoints; (2) insert the needles and stimulate to elicit response; (3) attach a small moxa stick to the needle tail and light the moxa stick; and (4) retain needles with moxa sticks for 30 min
Medicinal cake-separated moxibustion (medicinal cake + moxa cone)	
Acupoints	*Juque* (RN14), *Tianshu* (ST25, bilateral), *Pishu* (BL20, bilateral), *Xinshu* (BL15, bilateral), *Ganshu* (BL18, bilateral), *Shenshu* (BL23, bilateral)
Cake ingredients	*Dan Shen* (*Radix Salviae Miltiorrhizae*), *Shan Zha* (*Fructus Crataegi*), *Yu Jin* (*Radix Curcumae*), *Da Huang* (*Radix et Rhizoma Rhei*) and *Ze Xie* (*Rhizoma Alismatis*)
Cake preparation method	All cake ingredients in the same quantities were collected, ground into powder, mixed well with vinegar, and made into round, thin cakes of 1.5 cm in diameter, 3 mm in thickness and 3 g in weight; manufactured by Suzhou Medical Supplies Co., Ltd., Suzhou, China
Moxibustion specifications	Moxa cone, made of mugwort, with 1 cm in diameter; manufactured by Suzhou Medical Supplies Co., Ltd., Suzhou, China
Retention time	3 cones in total for 30 min
Manipulation	(1) locate the acupoints; (2) place the herbal cake on to the acupoints; (3) place a moxa cone onto the herbal cake and light the moxa cone; (4) renew the moxa cone once it is fully consumed, with 3 moxa cones in total per acupoint; and (5) retain herbal cakes with moxa cones for 30 min

### Discontinuity of intervention

Subjects in the study are able to withdraw from the study at any time, either partially or entirely.

When a subject fully consents to withdrawal from the study, the subject will no longer receive the investigational treatment and will have the right to discontinue any further involvement in the study, including any format of follow-up. Below is a list of reasons for discontinuing intervention:

- Withdrawal of full consent from subject
- Subject requests the ending of investigational intervention
- Administrative decision by the principal investigator
- Unanimous decision by the principal investigator/physician
- Pregnancy in a female subject
- Adverse event (e.g., serious adverse event related to the intervention)

### Concomitant medication

The following treatments are not permitted during the study:

- Prescribed lipid-regulating medications other than acupuncture and moxibustion or simvastatin, such as fibrates and derivatives, bile-acid sequestering resins
- Red yeast rice, niacin >200 mg per day, omega-3 fatty acid (e.g., DHA and EPA) >1000 mg per day. Any other drug that significantly affects lipid metabolism (e.g.,, systemic cyclosporine, systemic steroids (administered intravenously (IV), intramuscularly (IM), or per os (PO)), vitamin A derivatives and retinol derivatives for the treatment of dermatologic conditions (e.g.,, Accutane)). Vitamin A as part of a multivitamin preparation is permitted.
- Prescribed amphetamines, or amphetamine derivatives, and weight-loss medications

Besides, simvastatin or foods that are known potent inhibitors of CYP3A (itraconazole, ketoconazole, and other antifungal azoles, the macrolide antibiotics erythromycin, clarithromycin, and the ketolide antibiotic telithromycin, HIV protease inhibitors, the antidepressant nefazodone and grapefruit juice in large quantities (more than 1 quart daily)) should not be used during the study, because of their potential impacts on the metabolism of certain statins.

### Outcome measurements

#### Primary outcome

The primary outcome is the percentage change from baseline to the end of the study (after the 12-week intervention and after another 4-week follow-up) in LDL-C. The calculated LDL-C concentration will be determined at screening (entry to the study), day 1 (after TLC is completed and eligibility is determined), week 6, and week 12. LDL-C will be measured by preparative ultracentrifugation by the central laboratory. The LDL-C concentration calculated at screening will be reported only for the eligibility decision. The baseline lipid measurements for the purpose of analysis will be the day-1 lipid measurement after TLC (lipid stabilization). This will be collected after a ≥9-h fast and before receiving intervention.

#### Secondary outcomes

The secondary outcomes consist of three aspects: efficacy, safety and adherence. The efficacy outcomes include, from the baseline to the end of the study (after 12-week intervention and after another 4-week follow-up), the absolute change in LDL-C, the percentage change in HDL-C, the percentage change in total cholesterol (TC), the percentage change in TG, and the rate of subjects achieving LDL-C goal (see Table 5 in Appendix 1) [21]. The safety outcomes will be assessed by the physical examination, vital signs (including sitting blood pressure (BP) and heart rate (HR), electrocardiogram (ECG) and subject incidence of adverse events, after 12-week intervention and after another 4-week follow-up. The adherence outcome will be examined by the adherence rate, calculated as:

$$ \mathrm{Adherence}\ \mathrm{Rate}=\mathrm{Number}\ \mathrm{of}\ \mathrm{treatments}\ \mathrm{conducted}/\mathrm{Number}\ \mathrm{of}\ \mathrm{treatments}\ \mathrm{planned}. $$

### Study procedures

The study consists of four periods: Screening, TLC, Intervention, and Follow-up period. For the purpose of this study, a week is defined as seven calendar days. A month is defined as 28 days.

#### Screening

Screening is conducted among subjects who meet the inclusion criteria to determine the eligibility of subjects to enter the study. In order to determine the eligibility for the study, they will enter the screening process by signing and dating the Informed Consent Form for this study.

The following data will be obtained and procedures performed during initial screening:

- Written informed consent
- Medical history
- Vital signs (see Appendix 2)
- Review for adverse events (serious adverse events (SAEs) and study-related AEs are collected during screening)
- Concomitant therapy
- Physical examination
- 12-lead ECG in triplicate using centralized ECG services equipment
- Blood draw for fasting lipids, chemistry and serum pregnancy (women of childbearing potential only) by central laboratory (Appendix 2)

#### TLC period

Subjects who complete the screening successfully and who meet the inclusion/exclusion criteria will initiate therapeutic lifestyle change (TLC). The TLC adherence report form is made and sent to subjects to monitor their lifestyle change process. Re-assessment will be conducted after the TLC period.

#### Intervention period

##### Intervention day-1 visit

According to the re-assessment of fasting lipids after the TLC, a decision will be made on whether the lipid level has arrived at the target level and whether subjects should continue with the intervention. For those who have not reached the target lipid level, they will undertake randomization and receive first administration of the intervention and the date for that is considered to be day 1. For those who have reached the target lipid level after the TLC, they will be followed up for another 6 weeks and re-assessed for relative indexes in week 6.

##### Week 6 visit (±3 days)

Re-assessment on the study indicators of subjects will be conducted, including efficacy, safety, and adherence.

##### Week 12 end of study visit (±3 days)

Re-assessment on the study indicators of subjects will be conducted, including efficacy, safety, and adherence.

#### Follow-up period

A 4-week follow-up after treatment will be done in order to measure and monitor AEs and the effects of treatments at week 16. The study indicators of subjects will include efficacy and safety.

#### Trial flowchart

**Fig. 3:**
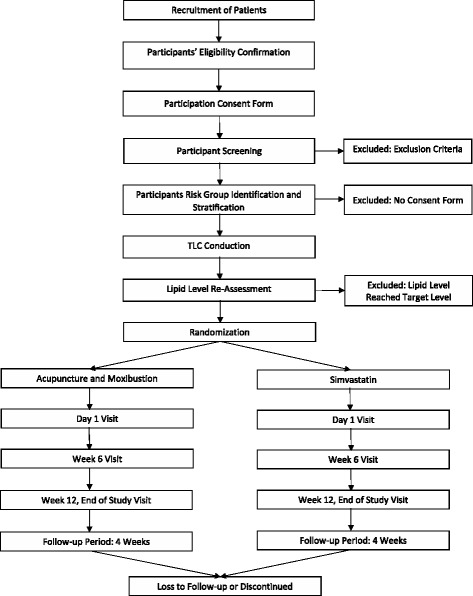
Trial flowchart. This figure provides an overview of participant flow in this trial

(Please refer to the supplementary document: Fig. 3. Trial flowchart)

### Sample size

A significance level of 0.95 and a power of 0.80 will be allocated for the calculation of sample size. In accordance with a previous study [17], we could employ an effect size of 0.343 on absolute value change of LDL-C value, and a standard deviation of 0.7. In this case we estimate that the sample size for the acupuncture group and active control group should be approximately 65, respectively. The total sample size needed is 130.

In case of missing observation and patients lost, we assume that 90% of subjects will remain in the study. Thus, the sample size for two groups should be roughly 70 for each group.

### Stratified and blocked randomization

According to the re-assessment of fasting lipid after the TLC, a decision will be made on whether the lipid level has arrived at the target level and whether subjects should continue with the intervention. Subjects will receive the intervention at the site at which they are recruited. Within each site, subjects will be stratified by risk groups (0–1 risk group, 2+ Risk Factor Group, and CHD or CHD risk equivalents group), as target lipid levels differ according to different risk groups. Subjects who have not reached the target lipid level after TLC, will be randomized to two arms at a ratio of 1:1 (acupuncture and moxibustion versus simvastatin). The randomization will be blocked (size = 4) to ensure that equal numbers of subjects are allocated to each arm. A computer-generated, blocked randomization sequence will be used to randomize subjects in the study. Assignment to the two treatment arms will be generated by the principal biostatistician using R (version 3.1.1; R Foundation for Statistical Computing, Austria. ISBN 3-900051-07-0) before the start of the study. The sequence will be held in a secure location in the hospital by the principal biostatistician; the researchers will be blinded to the number of cases in each randomization block and individual subject allocation will be conducted remotely via telephone based on the randomization sequence. Once a subject is eligible to receive the intervention, the principal biostatistician will be informed and will let physicians know each subject’s allocation by phone.

### Blinding

Due to the nature of the acupuncture and moxibustion interventions, neither participants nor care providers can be blinded to allocation and treatment stages, but are strongly inculcated not to disclose the allocation status of the participant at the follow-up assessments. The outcome assessment will be conducted by outcome assessors blind to the treatment allocation. In the stage of data analyses, an employee outside the research team will feed data into the computer in separate datasheets so that the data analysts can analyze the data without having access to information about the allocation.

To maintain the overall quality and legitimacy of the clinical trial, code breaks should occur only in exceptional circumstances when knowledge of the actual treatment is absolutely essential for further management of the patient. Investigators, including the outcome assessors and data analysts blind to treatment allocation, are encouraged to discuss with the medical advisor physicians if they believe that unblinding is necessary. The investigator is encouraged to maintain the blind as far as possible. The actual allocation must *not* be disclosed to the patient and/or other study personnel. There should not be any written or verbal disclosure of the code in any of the corresponding patient documents. The investigator must report all code breaks (with reason) as they occur on the corresponding Case Report Form page. Unblinding should not necessarily be a reason for study drug discontinuation.

### Data collection methods

The method of outcome data collection has been listed in previous parts. In order to ensure the data quality, each site’s personnel involved in this study will be trained to master the data collection requirements; the data to be collected and the procedures to be conducted at each visit will be reviewed in detail.

### Participant retention

In order to promote participant retention, once a patient is enrolled or randomized, the study sites will make every reasonable effort to follow the patient for the entire study period. In detail, study investigators and staff will:

- Maintain participants’ interests by materials and phone calls
- Provide periodic communications via materials and talks to inform the participants of our acknowledgement for their support
- Be as flexible as possible with the study schedule in resolving time conflicts with participants’ work and life

### Participant withdrawal

Participants will possibly withdraw from the study due to any reason at any time. The study investigators may also withdraw participants from the study with the purpose of protecting them and/or if they are unwilling to follow the study procedures. Once a withdrawal happens, it should be recorded by the steering committee and the data management team.

### Data management

#### Data forms and data entry

In this study, all data will be entered electronically. Original data along with the data collection form will be archived at the participating sites. Once a form has been filled out, the participating site staff will copy the form and send it to data management team for electronic re-entry.

Participant files will be stored by participation number. The storage location should be secured and accessible. The files will be kept in storage for 5 years after the study.

#### Data transmission and editing

Data transmission refers to the transmission of data from the original data form to computers used by the data management team. Data entry staff will carefully check whether the transmission is correct at the time of data entry. Data editing, either modification on single record or on multiple records, will be documented, along with the reason of the editing.

#### Data discrepancy reports and solutions

Errors may be detected by data analysts, including missing data error, or other specific errors in the data. The analyst should summarize the error and report to the data manager. The data manager who receives the data discrepancy reports will forward the specific problem to the data management staff in the participating site, retrieve the original data forms, and check whether there is an inconsistency.

There are several solutions for data discrepancy. If the data in the database is inconsistent with the original form, a primary choice is to correct the record in the database. Other alternative choices include missing data imputation techniques, data correction by checking other sources, and modifying the original data. The discrepancy reports and solutions should be documented for future reference.

#### Data back-up

The primary database will be backed up once per month. At the same time, the data analysis files will also be backed up.

### Statistical methods

#### Dataset analysis

For the primary endpoint of percentage change in LDL-C from baseline, the efficacy of acupuncture and moxibustion will be evaluated at week 12 by comparing the treatment effect to that of the active control group. A type I error of 0.05 will be allocated to the test.

There are three main analysis sets:

- Full analysis set (FAS): includes all randomized subjects who have received at least 1 investigational treatment
- Completer Analysis Set (CAS), includes subjects in the FAS who completed their scheduled intervention and have a nonmissing LDL-C value at week 12
- Effect Durability Analysis Set (EDAS), includes subjects in the FAS who completed their scheduled intervention and have non-missing LDL-C values at week 6 and week 12

#### Statistical analysis plan

The primary analysis set for the primary endpoint is the FAS. It is to use the repeated measures, linear mixed-effects model, including terms for treatment group, stratification factor, baseline LDL-C, scheduled visit and the interaction of treatment with scheduled visit. Missing values will not be imputed when the repeated measures, linear mixed-effects model is used.

We will use the chi-squared test for binary outcomes, and the *t* test for continuous outcomes. For subgroup analyses, we will use regression methods with appropriate interaction terms (respective subgroup treatment group). Multivariable analyses will be based on logistic regression for binary outcomes and linear regression for continuous outcomes. We will examine the residual to assess model assumptions and goodness-of-fit. For timed endpoints, such as mortality, we will use the Kaplan-Meier survival analysis followed by the multivariable Cox proportional hazards model for adjusting for baseline variables. We will calculate Relative Risk (RR) and RR Reductions (RRR) with corresponding 95% confidence intervals to compare dichotomous variables, and difference in means will be used for additional analysis of continuous variables. *P* values will be reported to four decimal places with *P* values < 0.05 reported as *P* < 0.05. Up-to-date versions of R (R Foundation) will be used to conduct analyses. For all tests, we will use two-sided *P* values with alpha no greater than 0.05 level of significance. We will use the Bonferroni method to appropriately adjust the overall level of significance for multiple primary outcomes, and secondary outcomes.

In investigation of the robustness of the analysis results, the primary analysis will be repeated using the CAS. In addition, parametric analysis of covariance (ANCOVA) and appropriate nonparametric methods will be used on the FAS, in which missing data will be imputed using the last-observation-carried-forward (LOCF) approach. The primary analysis will also be repeated for subgroups of interest.

#### Interim analyses

An interim-analysis is performed on the primary endpoint when 50% of patients have been randomized and have completed the interventions. The interim-analysis is performed by an independent statistician, blinded for the treatment allocation. The statistician will report to the independent data monitoring committee. The data monitoring committee will have unblinded access to all data and will discuss the results of the interim-analysis with the steering committee in a joint meeting. The steering committee decides on the continuation of the trial and will report to the central ethics committee.

#### Consistency analysis

To evaluate the consistency of the treatment effect of acupuncture and moxibustion, the following analyses will be performed:

- Consistency of treatment effects (acupuncture and moxibustion versus simvastatin) of week 6 and week 12: the 95% CI of the difference of the treatment effect at week 6 and the treatment effect at week 12 will be provided from the repeated measure mixed-effect model. The estimations will be from the FAS and the EDAS
- LDL change from week 6 to week 12:
  the treatment effect of percentage change from week 6 to week 12 will be estimated by using an ANCOVA model. The estimation of 95% CI will be based on the EDAS

Analysis of other secondary efficacy endpoints will be similar to the primary analysis of the primary endpoint. All tests will be based on a significance level of 0.05, without adjustment for multiple endpoints.

#### Safety summaries and adherence analysis

Safety summaries will include the subject incidence of AEs, summaries of laboratory parameters, vital signs, and ECGs.

### Data monitoring

A Data Monitoring Committee (DMC) has been established. The DMC is independent of the study organizers. During the period of recruitment to the study, interim analyses will be supplied, in strict confidence, to the DMC, together with any other analyses that the committee may request. This may include analyses of data from other comparable trials. In the light of these interim analyses, the DMC will advise the trial steering committee when:

- The active intervention has been proved, beyond reasonable doubt, to be different from the control (standard management) for all or some types of participants, and
- The evidence on the outcomes is sufficient to guide a decision from the health care providers

The Trial Steering Committee can then decide whether or not to modify intake to the trial. Unless this happens, however, the Steering Committee will remain ignorant of the interim results.

### Adverse events

An adverse event is defined as “any untoward medical occurrence in a clinical trial subject” [22]. An adverse event may or may not have a causal relationship with the treatment in the study. All adverse events observed by the investigator or study practitioner (e.g., physicians), or reported by the subjects, are to be recorded in the medical record of the subject. If a pre-existing medical condition of the subject worsens, this is considered one type of adverse event. For example, if a patients’ diabetes, migraine headaches or gout worsens in time (e.g., increased in severity, frequency of duration) with the administration of the treatment, this indicates that the administration of the treatment may be associated with a significant worse outcome in the subject.

All adverse events observed by the investigator or study practitioner (e.g., physicians) or reported by the subject after randomization through the end of study will be reported by the investigator to the applicable electronic Case Report Form (eCRF) (e.g., Adverse Event Summary eCRF).

#### Adverse event attributes

Below are the attributes the investigator must assign to each adverse event:

- Adverse event diagnosis or syndrome(s), if known (if not known, signs or symptoms),
- Dates of onset and resolution,
- Severity (and/or toxicity per protocol)
- Assessment of relatedness to investigation treatment, and
- Action taken

#### Adverse event assessment

Below is a list of questions the investigators must assess. Each question is answered with either a “yes” or “no”:

- Is there a reasonable possibility that the event may have been caused by the study intervention?
- Is there a reasonable possibility that the event may be related to screening procedures?
- Is there a reasonable possibility that the event may have been caused by a study activity/procedure?

#### Adverse events and laboratory tests

Laboratory tests results will be reviewed thoroughly by the investigator to determine whether the change in abnormal values from the baseline values is clinically significant (based on clinician’s own judgment). After reviewing the changes, the investigator will determine whether an adverse event will be reported.

- Abnormal laboratory findings that are not clinically significantly different from baseline values will not be recorded as adverse events
- Laboratory findings that are clinically significant, that require treatments or adjustments from the current treatment will be recorded as adverse events
- Clinically sequelae should also be recorded as the adverse event, where applicable

#### Adverse events and participant withdrawal

The investigator will use his/her clinical judgment to determine whether a subject should be removed from the study due to adverse events. The subject or their legal representatives also have the full right to withdraw from the treatment due to an adverse event or concerns for an adverse events. However, the investigator will encourage the subjects to undergo at least an end of the study assessment.

### Serious adverse events

#### Definition

A serious adverse event is defined as an adverse event that meets at least one of the following criteria:

- Fatal
- Life-threatening (places the subject at immediate risk of death)
- Requires in-patient hospitalization or prolongation of existing hospitalization, (the criterion of “requires hospitalization” for an adverse event, indicates an event necessitated an admission to a health care facility, e.g., overnight stay in hospital)
- Results in persistent or significant disability/incapacity
- Congenital anomaly/birth defect
- Other medically important serious event

A serious adverse event can still be recorded if an investigator considers an event to be clinically significant. In this case, the event will be classified as “other medically important serious event” and comprehensive documentation of the events’ severity will be recorded on the subject’s medical record. Some examples of this include: allergic bronchospasm, convulsions, blood dyscrasia, or events that necessitate an emergency room visit, outpatient surgery, or urgent intervention.

#### Reporting procedures for serious adverse events

All observed or reported serious adverse events after randomization through 4 weeks after the last investigational treatment will be recorded on the subjects’ medical records by the investigator and submitted to the principal investigator within one working day of discovery of the adverse event. These include: new information reported on a previously known serious adverse event, serious adverse event possibly related to the treatment, and full withdraw by the subject from the study due to serious adverse event.

In the case of a serious, unexpected, and related adverse event, the subject may be unblinded by the investigator prior to submission of the adverse event to the regulatory authority. Investigators will receive information on how to report adverse serious events to authorities in accordance to local requirements and Good Clinical Practice prior to the clinical trial. All serious adverse events will be reported to appropriate Independent Ethics Committee (IEC).

### Pregnancy reporting

If a pregnancy occurs during the time when the female subject is undertaking the study intervention, the pregnancy should be reported to the research team. Pregnancy after undertaking the study intervention for an additional 4 weeks should also be reported.

## Study dissemination

### Confidentiality

All study-related information will be stored securely at the study site. All participant information will be stored in locked file cabinets in areas with limited access. All laboratory specimens, reports, data collection, process, and administrative forms will be identified by a coded ID (identification) number only to maintain participant confidentiality. All records that contain names or other personal identifiers, such as locator forms and Informed Consent Forms, will be stored separately from study records identified by code number. All local databases will be secured with password-protected access systems. Forms, lists, logbooks, appointment books, and any other listings that link participant ID numbers to other identifying information will be stored in a separate, locked file in an area with limited access. Participants’ study information will not be released outside of the study without the written permission of the participant.

### Access to data

The Data Management Coordinating Center will oversee the intrastudy data sharing process. All data sets will be password-protected. The principal investigator will have direct access to the data sets. With her permission, the statistician will utilize the data sets for analyses. Requests from outside for further research cooperation will be discussed by the Steering Committee. The research protocol will be published. There is no plan for granting public access to the full participant-level data sets.

### Publication policy

All publications should be conducted and monitored by the principal investigator and the Steering Committee. That means that each paper or abstract must be written, reviewed, released, and published with the authorization of the PI and the Steering Committee. The timing of presentation of the endpoint data and the meetings at which they may be presented will also be determined by PI and the Steering Committee.

In order to achieve the study objectives and maintain scientific integrity, data should be analyzed collectively; a participating site is not permitted to publish its data and analysis results independently. If there are some invitations from workshops, symposia, and volumes in related area, individuals who work on such requests should report to the PI and Steering Committee with a detailed proposal, and state clearly what to present and how to present.

### Authorship eligibility

Many topics will be suggested based on the study database, and each topic might be possible to be developed into a peer-reviewed article. The PI and the Steering Committee will work together to decide the authorship of each article, on basis of contributions to the manuscripts; the investigator who contributes most will be considered as the first author. Disputes regarding authorship will be resolved by the PI with careful considerations. If professional writers are needed, the PI and the Steering Committee will determine whether to include them in authorship.

## Discussion

The current conventional treatment on hyperlipidemia, statins, not only can entail significant cost to patients and society, but also is associated with low treatment compliance and high rates of side effects [3, 4]. Effective treatment alternatives on hyperlipidemia have been explored to address the shortcomings of statins; and acupuncture and moxibustion are considered as potentially effective regimens based on clinical practices and clinical research [9–11, 17, 18, 23]. However, acupuncture and moxibustion, separately, have not been proved to be as effective as statin from previous studies, despite the existence of their clinical effects [9–11, 17, 18, 23]. Additionally, as approaches of complementary and alternative medicine (CAM), their introduction to the mainstream medical community is often limited due to lack of robust evidence and differences in the philosophy adopted. Therefore, the RCT described in this paper has been developed to validate the efficacy and safety of combined acupuncture and moxibustion on hyperlipidemia via a well-designed evaluation.

There are several strengths in design of this study. First, the intervention adopted in this study is developed not only based on profound TCM theory but also supported by comprehensive scientific research. The theory guiding the acupoints selection and traditional Chinese herb composition has been studied, identified and well-demonstrated in this study which is discussed in the “Treatment rationale” section. Meanwhile, several studies from the perspectives of laboratory animal medicine and clinical medicine have been conducted [9–11, 17, 18, 23] which serve as the key foundation of intervention development in this study. Specifically, the biological mechanism was explored on how cake-separated moxibustion can have an impact upon hyperlipidemia; and the acupoint selection and regimen implementation from ample TCM theory were validated in multiple clinical studies.

Second, the TLC will be included prior to both interventions of acupuncture and moxibustion and statins, which enables this study to reflect the actual practice in clinical settings to the greatest extent. According to the guideline of hyperlipidemia treatment [20], TLC is recommended as the first step before any other treatment. By adhering to the commonly adopted clinical guideline during study procedure, on the one hand, we can take good care of the “non-maleficence” and “justice” principles in study ethics, because the opportunity of receiving appropriate medication will not be compromised for patients attending this study; on the other hand, this can enhance the generalizability of this study due to following the clinical operations in real life.

Third, the stratification on risk levels for heart disease [19, 20] will be applied to this study, which makes possible that the effect of interventions of this study can be differentiated and specified according to different risk groups. Patients with hyperlipidemia may enter the study with different initial lipid levels, due to different time points of initiating medication intervention for groups with different risk levels. Meanwhile, the goal lipid level for each patient may also differ accordingly. It is impractical and erroneous to set exactly the same criteria in determining the clinical effect for all the patients. In recognition on this, patients will be stratified by their risk levels during the study, and then the effects of acupuncture and moxibustion versus simvastatin can be observed and examined within different risk levels, instead of within a large group with extensive variance. This stratification reflects that patient’s risk levels for heart diseases are considered important impacting factors on the outcomes in this study; it enables exploring this impact upon the effects of interventions, which can further guide the appropriate intervention by targeting on the right populations. In addition, different treatment goals will be adopted for different risk groups according to the standard clinical guideline; this ensures the study will be implemented in accordance with the daily clinical practices to the most extent, thus, the external validity can be mostly warranted.

However, certain limitations still exist in this study. One is the absence of a placebo control group due to the limited study budgets. Because of this lack of comparison, it may not be able to determine whether the outcome observed is due to the therapeutic effect of acupuncture and moxibustion or its placebo effect, if acupuncture and moxibustion is proved to be inferior to simvastatin. Nevertheless, judgment from clinical experiences and relevant findings from previous studies can help in identifying and justifying the effect with clinical significance.

Another limitation is the absence of blinding in patients and health care providers due to the operation characteristics of acupuncture and moxibustion. With patients knowing their intervention assignments after randomization, there may be a potentially higher possibility of cross-over between and drop-off from these two arms; thus, the potential selection bias may occur. But detailed explanation can be given to patients on these two arms of interventions in order to inform potential effects and safety in both arms. This will help limiting the drop-off and cross-over rates. Moreover, with the intervention assignment revealed, the effect identified from the acupuncture and moxibustion group may not fully result from its therapeutic aspect but a psychological aspect. But the outcome measures examined in this study are all objective – based on the fasting lipid level; and in the analysis process intervention assignment is fully blinding to data assessors. Therefore, the information bias due to lack of blinding could be controlled to a considerable extent.

## Conclusion

In conclusion, efforts have been devoted, in this study, to developing a well-designed assessment approach to evaluate the efficacy and safety of combined acupuncture and moxibustion on treating hyperlipidemia, by integrating the TCM regimens with the standardized Western medicine appraisal approach. It is expected this assessment will introduce a potentially effective alternative treatment of hyperlipidemia by borrowing the wisdom from the time-tested practice approach, TCM.

## Trial status

- Primary registry and trial identifying number: ClinicalTrials.gov NCT02269046
- Date of registration in primary registry: 16 October 2014
- Primary sponsor: The First Affiliated Hospital of Hunan University of Traditional Chinese Medicine
- Contact for public queries: Dr. Mailan Liu. Email: 445007305@qq.com
- Contact for scientific queries: Dr. Xiaorong Chang. Email: xrchang1956@163.com
- Public title: Acupuncture and Moxibustion for Hyperlipidemia
- Scientific title: Acupuncture and Moxibustion for Hyperlipidemia (AMH-RCT): Study Protocol for a Randomized Controlled Trial
- Countries of recruitment: China
- Health condition(s) or problem(s) studied: hyperlipidemia
- Intervention(s): acupuncture and moxibustion, simvastatin
- Study type: interventional
- Date of first enrollment: December 2014
- Target sample size: 130
- Recruitment status: recruiting

## Appendix 1

**Table 2:** Major risk factors for CHD other than LDL [13]

Cigarette smoking	
Hypertension (BP ≥140/90 mmHg or on antihypertensive medication)	
Low HDL cholesterol (<40 mg/dL)	
Family history of premature CHD (CHD in male first degree relative <55 years; CHD in female first degree relative <65 years)	
Age (men ≥45 years; women ≥55 years)

**Table 3:** CHD and CHD equivalents

• Other clinical forms of atherosclerotic disease (peripheral arterial disease, abdominal aortic aneurysm, and symptomatic carotid artery disease)	
• Diabetes	
• Multiple risk factors that confer a 10-year risk for CHD >20%

**Table 4:** Nutrient Composition of the TLC Diet

Nutrient	Recommended Intake
Saturated fat^a^	Less than 7% of total calories
Polyunsaturated fat	Up to 10% of total calories
Monounsaturated fat	Up to 20% of total calories
Total fat	25-35% of total calories
Carbohydrate^b^	50-60% of total calories
Fiber	20-30 g/day
Protein	Approximately 15% of total calories
Cholesterol	Less than 200 mg/day
Total calories (energy)^c^	Balance energy intake and expenditure to maintain desirable body weight/prevent weight gain

^a^Trans fatty acids are another LDL-raising fat that should be kept at a low intake

^b^Carbohydrate should be derived predominantly from foods rich in complex carbohydrates including grains, especially whole grains, fruits, and vegetables

^c^Daily energy expenditure should include at least moderate physical activity (contributing approximately 200 Kcal per day)

**Table 5:** Three Categories of Risk that Modify LDL-C Goals

Risk Category	LDL Goal (mg/dL)
CHD and CHD risk equivalents	<100
Multiple (2+) risk factors	<130
0 to 1 risk factor	<160

## Appendix 2 Measurement of Lipid level and Vital Signs

### Measurement of Vital Signs

Blood pressure (BP) and heart rate (HR) should be measured at each visit. BP should continue to be measured in the same arm as in the parent study unless a concomitant condition favors the use of a different arm. The appropriate size cuff should be used.

The diastolic blood pressure (DBP) will be recorded as the pressure noted when sound disappears (Korotkoff Phase V). BP and HR measurements should be determined after the subject has been seated for at least 5 minutes. The subject’s pulse should be measured for 30 seconds and the number should be multiplied by 2 to obtain heart rate.

### Lipid Measurements

The baseline lipid measurements for the purpose of analysis will be the central laboratory screening and day 1 lipid measurement collected after a ≥ 9 hour fast and before receiving intervention. At each time points, calculated LDL-C concentration will be determined. In addition, at screening, day 1, week 6, and week 12 LDL-C will be measured by preparative ultracentrifugation by the central laboratory. Only the screening calculated LDL-C concentration will be reported to the site for the eligibility decision. For subjects who are rescreened, data from the first screening period will not be used for the analysis. Investigators, subjects, and the study team will be blinded to post-randomization central laboratory lipid panel values for the duration of the study until unblinding of the database. In addition, investigators and staff involved with this trial and all medical staff involved in the subject’s medical care should refrain from obtaining lipid panels between randomization and at least 4 weeks after the subject ends the study at week 12 (to avoid potential unblinding). If a lipid panel is drawn, all reasonable steps must be undertaken to avoid informing the subject and study personnel of the results.

### Laboratory Assessments

All screening and on-study laboratory samples will be processed and sent to the central laboratory.

The central laboratory will provide a study manual that outlines handling, labeling, and shipping procedures for all blood samples. The date and time of sample collection will be recorded in the source documents at the site.

Table below outlines the specific analytic for serum chemistry, hematology, urinalysis, and other testing to be conducted.

**Table:**

Chemistry	Coagulation	Urinalysis	Hematology	Other Labs
Sodium Potassium Chloride Bicarbonate Total protein Albumin Calcium Magnesium Phosphorus Glucose (Fasting) BUN or Urea Creatinine Uric acid Total bilirubin Direct bilirubin CK ALP LDH AST (SGOT) ALT (SGPT)	PT/INR	Specific gravity pH Blood Protein Glucose Bilirubin WBC RBC Epithelial cells Bacteria Casts Crystals	Hemoglobin Hematocrit MCV MCH MCHC RDW Platelets WBC Differential • Neutrophils • Bands • Eosinophils • Basophils • Lymphocytes • Monocytes	Fasting lipids • Total cholesterol • HDL-C • LDL-C • Triglycerides • VLDL-C ApoA1 ApoB hsCRP Lp(a) Anti-AMG 145 antibodies PK • AMG 145 • PCSK9 HbA1c FSH (if needed per exclusion 4.2.17) TSH eGFR (calculated) Fasting Vitamin E

## Additional file

Additional file 1: SPIRIT 2013 Checklist. (DOC 121 kb)

## Abbreviations

AE: Adverse event
AHA: American Heart Association
ALP: Alkaline phosphatase
ALT: Alanine aminotransferase
AMH: Acupuncture and Moxibustion for Hyperlipidemia
ANCOVA: Analysis of covariance
ApoA1: Apolipoprotein A-1
ApoB: Apolipoprotein B
AST: Aspartate aminotransferase
BL: Bladder meridian
BP: Blood pressure
CABG: Coronary artery bypass graft
CAS: Complete analysis set
CHD: Coronary heart disease
CI: Confidence interval
CK: Creatine kinase
CVD: Cardiovascular disease
DBP: Diastolic blood pressure
DHA: Docosahexaenoic acid
DMC: Data monitoring committee
ECG: Electrocardiogram
EDAS: Effect durability analysis set
eGFR: Estimated glomerular filtration rate
EPA: Eicosapentaenoic acid
FAS: Full analysis set
HDL-C: High density lipoprotein cholesterol
HR: Heart Rate
hsCRP: High sensitivity C-reactive protein
IEC/IRB: Independent Ethics Committee/Institutional Review Board
LDH: Lactate dehydrogenase
LDL-C: Low-density lipoprotein cholesterol
LLN: Lower limit of normal
LOCF: Last observation carried forward
Lp(a): Lipoprotein(a)
NCEP ATP III: National Cholesterol Education Program Adult Treatment Panel III
NYHA: New York Heart Failure Association
PC: Pericardian meridian
PCI: Percutaneous coronary intervention
PPAR: Peroxisome Proliferator-Activated Receptor
RBC: Red blood cells
RCT: Randomized Controlled Trial
RN: Ren meridian
RR: Relative Risk
RRR: RR reductions
SAE: Serious adverse event
SBP: Systolic blood pressure
SP: Spleen meridian
ST: Stomach meridian
TC: Total cholesterol
TCM: Traditional Chinese Medicine
TG: Triglycerides
TLC: Therapeutic Lifestyle Change
TLR: Toll-like receptor
TLR4: Toll-like receptor 4
TSH: Thyroid Stimulating Hormone
ULN: Upper limit of normal
VLDL-C: Very low-density lipoprotein cholesterol
WBC: White blood cell
